# Size dependent translocation and fetal accumulation of gold nanoparticles from maternal blood in the rat

**DOI:** 10.1186/s12989-014-0033-9

**Published:** 2014-09-10

**Authors:** Manuela Semmler-Behnke, Jens Lipka, Alexander Wenk, Stephanie Hirn, Martin Schäffler, Furong Tian, Günter Schmid, Günter Oberdörster, Wolfgang G Kreyling

**Affiliations:** 1 Institute of Lung Biology and Disease, Helmholtz Zentrum München ¿ German Research Center for Environmental Health, Neuherberg/Munich, 85764, Germany; 2 Department of Environmental Medicine, University of Rochester, Rochester, New York, USA; 3 Institute of Inorganic Chemistry University Duisburg-Essen, Essen, 45117, Germany; 4 Institute of Epidemiology II, Helmholtz Zentrum München ¿ German Research Center for Environmental Health, Neuherberg/Munich, 85764, Germany; 5 Current address: Bavarian Health and Food Safety Authority, Oberschleissheim, 85764, Germany; 6 Current address: Walter Brendel Centre of Experimental Medicine, Ludwig-Maximilians-Universität München, Munich, Germany; 7 Current address: Focus Research Institute, Dublin Institute of Technology, Dublin, Ireland

**Keywords:** Gold nanoparticles, Accumulation in rat fetus, Placenta, Transtrophoblastic channel, Amniotic membrane

## Abstract

**Background:**

There is evidence that nanoparticles (NP) cross epithelial and endothelial body barriers. We hypothesized that gold (Au) NP, once in the blood circulation of pregnant rats, will cross the placental barrier during pregnancy size-dependently and accumulate in the fetal organism by 1. transcellular transport across the hemochorial placenta, 2. transcellular transport across amniotic membranes 3. transport through ~20 nm wide transtrophoblastic channels in a size dependent manner. The three AuNP sizes used to test this hypothesis are either well below, or of similar size or well above the diameters of the transtrophoblastic channels.

**Methods:**

We intravenously injected monodisperse, negatively charged, radio-labelled 1.4 nm, 18 nm and 80 nm ^198^AuNP at a mass dose of 5, 3 and 27 ?g/rat, respectively, into pregnant rats on day 18 of gestation and in non-pregnant control rats and studied the biodistribution in a quantitative manner based on the radio-analysis of the stably labelled ^198^AuNP after 24 hours.

**Results:**

We observed significant biokinetic differences between pregnant and non-pregnant rats. AuNP fractions in the uterus of pregnant rats were at least one order of magnitude higher for each particle size roughly proportional to the enlarged size and weight of the pregnant uterus. All three sizes of ^198^AuNP were found in the placentas and amniotic fluids with 1.4 nm AuNP fractions being two orders of magnitude higher than those of the larger AuNP on a mass base. In the fetuses, only fractions of 0.0006 (30 ng) and 0.00004 (0.1 ng) of 1.4 nm and 18 nm AuNP, respectively, were detected, but no 80 nm AuNP (<0.000004 (<0.1 ng)). These data show that no AuNP entered the fetuses from amniotic fluids within 24 hours but indicate that AuNP translocation occurs across the placental tissues either through transtrophoblastic channels and/or *via* transcellular processes.

**Conclusion:**

Our data suggest that the translocation of AuNP from maternal blood into the fetus is NP-size dependent which is due to mechanisms involving (1) transport through transtrophoblastic channels ¿ also present in the human placenta ¿ and/or (2) endocytotic and diffusive processes across the placental barrier.

## Background

The multiple benefits associated with the increasing manufacturing of nanotechnology based products are met with equally increasing concerns about potential adverse health effects from exposure of consumers to engineered nanoparticles (NP) [1]. These concerns are based on findings from biokinetic studies in humans and experimental animals revealing that NP may enter the body *via* the respiratory tract or the gastro-intestinal-tract and thereby be distributed throughout the body [1]-[6]. However, although translocation of NP from the portal of entry across cellular barriers (*e.g*., alveolo-capillary barrier) has been described, the amount of NP reaching the blood circulation from the primary portal of entry appears to be rather low [5]-[11]. However, the small fractions of insoluble NP translocating into systemic circulation localize and accumulate in secondary organs such as liver, spleen, heart and others as well as in bone marrow during chronic exposure [12],[13]. One of the critical protective barriers is the placenta, providing protection of the unborn from potential toxicants. It is known, though, that the placental barrier function cannot be complete because the fetus requires continuous transfer of glucose and nutrients, including proteins, phospholipids, antibodies, and hormones from the maternal blood circulation [14]. Therefore, even air pollutants, side stream smoke and engineered particulates, after reaching the blood compartment, may gain access to the fetus and potentially cause adverse effects *in utero* or postnatally [15]-[18]. Previous mouse exposure studies with Diesel exhaust during pregnancy supported a role of particulate air pollution upon adverse health effects in the central nervous system of the offspring [19]-[21]. Recently translocation of 50¿250 nm polystyrene particles across human term placentas [22] was shown while no measurable translocation of 15 and 30 nm poly-ethylene-glycol coated AuNP was observed in a similar *ex vivo* model [23]. Indeed, a recent paper by Yamashita [24] reported size-dependent translocation from mouse placenta into fetuses following very high doses (800 ?g/mouse) of SiO_2_ NP (70 nm) and TiO_2_ NP (35 nm) administered by intravenous (IV) injection. Based on this study a commentary by Keelan [25] raised a number of questions such as ¿Whether size-dependent effects observed ¿ reflect size inherent exclusion property of the placenta itself or a characteristic of the specific nanomaterial investigated in the Yamashita study¿; and ¿the mechanisms responsible which transported NP from within the trophoblast layers into the fetal circulation are still unclear¿. A just recently published paper reported on the translocation of IV injected 20 nm and 50 nm AuNP (stabilized in citrate and suspended in saline at a dose of 50 ?g/mouse) into the placenta of pregnant mice at gestation days 16 or 17 [26]. AuNP of both sizes were observed in maternal liver and the placenta but not in the fetal liver. Additional results of immunoreactivity tests suggested that IV administration of AuNP may upregulate clathrin- and caveolin-mediated endocytosis at the maternal¿fetal barrier in the mouse placenta.

**Figure 1:**
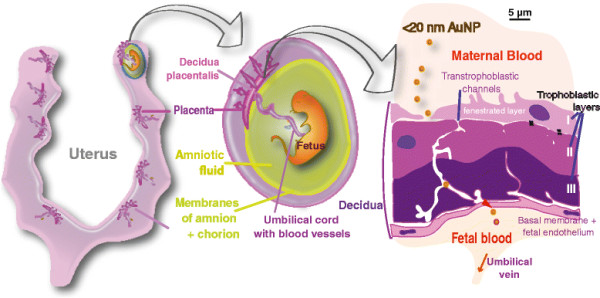
**Schematics of potential NP transport including the uterine wall, the amniotic membrane enclosing the individual fetus and the placental barrier; potential NP transport pathways: (1) across the trophoblastic layers and through their transtrophoblastic channels within the placenta and (2) across the uterine wall and amniotic membrane.** Hemotrichorial rat placentas are reported to have transtrophoblastic canaliculi of about 20¿25 nm diameter through the trophoblastic layers II and III connecting maternal and fetal blood across the placental barrier [29],[31]. There is convincing evidence that these channels are continuous [30],[33] and are also present in the human placenta. At gestation day 18?±?1 the yolk sac has considerably shrunk and is not shown in these schematics.

Given the concerns about potential adverse health effects of NP and their demonstrated - albeit limited - propensity to cross cell barriers, we wanted to determine as to whether realistic, low doses of NP, once in the blood circulation, will cross placental barriers during pregnancy and accumulate in fetuses. The possible mechanisms and pathways to cross the placental barrier include simple diffusion or pinocytosis *via* clathrin, megalin or caveolin mediated transport [27],[28]; and also *via* transtrophoblastic channels (canaliculi) of about 20¿25 nm diameter that connect maternal blood across the hemochorial placenta of humans and rats directly to the fetal blood [29]-[31]. We hypothesize that these transtrophoblastic channels represent a pathway for NP in a size dependent manner from the placenta to fetal circulation in addition to endocytotic and diffusive transport mechanisms. In order not to overwhelm the body and its responses by irrelevantly high doses we used only small amounts of AuNP radioactively labelled with tracer amounts of ^198^Au (^198^AuNP). We intravenously injected monodisperse, negatively charged, insoluble AuNP of three well-separated sizes: either well below (1.4 nm), or of similar size (18 nm) or well above (80 nm) the 20¿25 nm size of the transtrophoblastic channels; all three AuNP were coated by ionic ligand molecules of sulfonated triphenylphosphine (S-TPP). Moreover, the selected AuNP sizes are good representatives for the entire NP range. In addition, ?g-range AuNP doses ¿ although administered as a bolus - were chosen because there is sufficient evidence that toxic responses in the mother¿s body and in the fetuses are very unlikely at these rather low doses [32]. This prove-of-principle study was based on the determination of quantitative AuNP distribution in the entire organism of pregnant rats including placental and fetal tissues, see schematics in Figure 1.

### Animal model and hypothesis

We used the pregnant rat model on day 18?±?1 of gestation to assess *in vivo* the concept of ^198^AuNP translocation across the placenta. The barrier between fetal and maternal part of the late stage placenta is only a few micrometer thick allowing for the exchange of nutritions and fluids [31],[34]. The hemotrichorial anatomy of the rat placenta allows the maternal blood to be in direct contact with the chorion. The outer trophoblastic layer of the rat placenta is fenestrated so ^198^AuNP could enter into the labyrinth between the fenestrated outer layer and the middle trophoblastic layers of the placenta by diffusion (Figure 1). We hypothesize that in addition to diffusion also/or endocytotic processes may play a role in the transport of ^198^AuNP across the placental barrier [26]. These include pinocytotic or receptor mediated processes which are functional for nutrition and supply [31],[34]. Yet, no experimental data on these transcellular pathways exist [35]. This pathway involves sequential transcellular passages through the trophoblastic cell layers of the labyrinth-type hemotrichorial rat placenta which is likely to increase the time of translocation and cellular retention of AuNP. Furthermore, hemotrichorial rat placentas have transtrophoblastic canaliculi of about 20¿25 nm diameter through the trophoblastic layers II and III (Figure 1) connecting maternal and fetal blood across the placental barrier [29]-[31].

We suggest that 1.4 nm ^198^AuNP pass easily through these canaliculi while only a smaller fraction of 18 nm ^198^AuNP translocate *via* these canaliculi, in contrast to large 80 nm ^198^AuNP which will not be able to reach the fetal circulation by this pathway. Furthermore, based on the results obtained from the three different sized ^198^AuNP, we are able to estimate the contribution of transcellular translocation processes like endocytosis and exocytosis across the uterine and amniotic membranes as well as across the hemotrichorial trophoblastic layers.

### Role of the yolk sac

While the yolk or vitelline sac provides most of the nutrition for the rat fetuses during the early gestational stage it does no longer play a significant supply function on day 18?±?1 of gestation, even though it is still present but at a much smaller size. On day 18 the rat placenta functions are fully optimized to support the development of the growing fetuses in their individual amnions. No data exist for fetal translocation of NP *via* the vitelline sac; however, even if existent, it is most likely minimal when considering the small surface area of its membrane or the amniotic fluid.

### 8AuNP dose considerations

By using radioactive ^198^Au-labelling of the AuNP it was possible to detect anticipated tracer amounts in the placental and fetal samples of a few ng of ^198^AuNP following IV injection of low doses which should not result in a bolus overload effect. AuNP were neutron-activated prior to use only to the extent required to detect the ^198^AuNP in the placental and fetal samples ¿ see Methods. As a result the delivered radio-dose of about 100 kBq of the short-lived ^198^Au radio-isotope (half-life 2.7d) is far below from causing any acute radio-toxic effect during 24 hours retention time ¿ even on a nanoscopic level in the direct vicinity of these ^198^AuNP: the individual 1.4 nm and 18 nm ^198^AuNP contain maximally one ^198^Au atom and the 80 nm ^198^AuNP contain on average 20 ^198^Au atoms. Because the radio-isotope ^198^Au is chemically the same as in the AuNP core there is no leaching of the radio-label out of the insoluble matrix of AuNP. Yet, the selection of such rigid experimental parameters has some limitations regarding the visualisation within the tissues: For the tiny 1.4 nm AuNP clusters consisting of 55 Au atoms in stable configuration there is currently no imaging technology for biological tissues to identify them. (Imaging requires carefully cleaned and specially prepared substrate surfaces.) Even silver enhancement does not work for AuNP?<?2 nm [36].

Likewise, the anticipated low amount of tracer results in such a low number of the largest 80 nm AuNP to be expected in the placenta that they are practically not detectable by electron microscopy. For example, in order to detect 30 AuNP of 80 nm size an estimated number of 2 × 10^4^ pieces of 3 × 3 mm^2^ × 80 nm fetal tissue slices would be required for detection by electron microscopy screening which is unfeasible with current technology.

## Results and discussion

Physico-chemical properties of monodisperse, negatively charged, insoluble and radio-labeled ^198^AuNP are given in the Methods section and have been described previously [5],[6],[10],[11]. In addition, we provide *in vivo* data in the Supporting Information suggesting rapid replacement of the ionic sulfonated triphenylphosphene (S-TPP) surface modification from the AuNP after IV injection.

### Extra-uterine 198AuNP biodistribution

**Figure 2:**
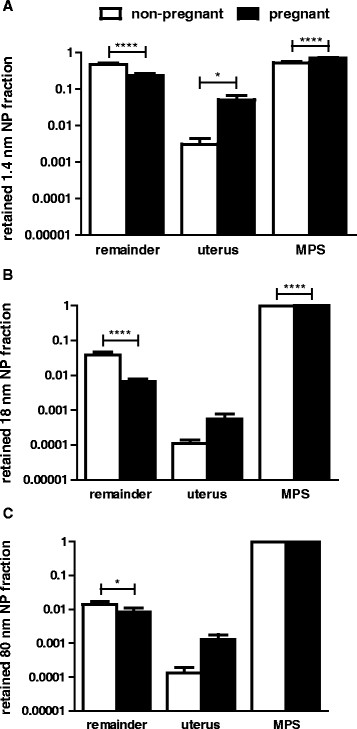
**Comparison of pregnant versus non-pregnant rats: comparison of 24-hour retained**^**198**^**AuNP fractions relative to the initially administered dose in either pregnant rats in their 3**^**rd**^**trimester or non-pregnant controls: upper panel 1.4 nm AuNP; middle panel 18 nm AuNP; lower panel 80 nm AuNP.** Note that fractions are ^198^Au radioactivity- and, hence, Au-mass based. Compartments are the **(A)** mononuclear phagocytic system (MPS consisting of liver, spleen, lungs), **(B)** the remaining carcass (remainder) including skeleton, soft tissues, skin and all other organs and **(C)** the uterus with or without the progeny. MPS and remainder samples were corrected for ^198^AuNP in the remaining blood. Note the uterus of the pregnant rats comprises of the uterine walls, the placentas and all amnions with fetuses. Data are given as fractions of the intravenously injected ^198^AuNP doses. (n?=?4; * p?<?0.05; ** p?<?0.01; *** p?<?0.001; **** p?<?=0.0001). Statistical analysis by one-way analysis of variance (ANOVA) followed by post hoc Sidak¿s multiple comparisons test.

As shown previously in non-pregnant female rats we found prominent ^198^AuNP uptake and retention in the liver 24 h after IV injection of ^198^Au labelled AuNP [6],[10]. The retained fraction of IV administered 18 nm or 80 nm ^198^AuNP in mononuclear phagocyte system (MPS) (here represented by liver, spleen and lungs) was greater than 0.97 in pregnant and non-pregnant rats (Figure 2); note fractions are ^198^Au radioactivity and, hence, mass based. These fractions were dominated by AuNP retention in the liver. Retention in all other organs and tissues did not differ significantly between pregnant and non-pregnant rats for both, 18 nm and 80 nm ^198^AuNP. In contrast, we found a fraction of 0.52?±?0.04 of the administered 1.4 nm ^198^AuNP in the MPS of non-pregnant rats and a significantly higher fraction of 0.71?±?0.02 in the MPS of pregnant rats, respectively. Both fractions were dominated by AuNP retention in the liver (0.50?±?0.03 and 0.68?±?0.02, respectively). Interestingly, lower lung retention and higher liver retention were significantly different (p?<?0.0001) for pregnant rats when compared to non-pregnant rats, but there was no difference in spleen retention. A more detailed discussion is given in Additional file 1. Additionally, 1.4 nm ^198^AuNP retention in kidneys, heart and skin and remaining carcass of pregnant rats were significantly (p?<?0.0001) lower. Carcass consisted of skeleton and soft tissues (muscles and fat); yet, a sample of muscle and humerus did not significantly vary between pregnant and non-pregnant rats.

Retention of the administered 1.4 nm, 18 nm and 80 nm ^198^AuNP was significantly lower in the remainder of pregnant rats compared to non-pregnant rats. As detailed in Additional file 1 urinary excretion of 18 nm and 80 nm ^198^AuNP was negligible in non-pregnant rats; but about half of the of 0.05 ¿ 0.1 excreted fraction of 1.4 nm ^198^AuNP were found in urine in both pregnant and non-pregnant rats indicating renal filtration of a few percent of the injected 1.4 nm ^198^AuNP. Fecal excretion in both pregnant and non-pregnant rats resulted from hepato-biliary AuNP clearance [6] and shows strong inverse size dependency, Additional file 1: Figure S4. For all three ^198^AuNP, the hepato-biliary AuNP clearance was significantly reduced in pregnant rats compared to non-pregnant controls, which is discussed in more detail in the Additional file 1. These differences indicate that the altered physiology of the pregnant rat significantly affects AuNP biokinetic, a finding that requires follow-up studies to identify underlying mechanisms.

### Intra-uterine 198AuNP biodistribution in pregnant rats

Twenty-four hours after a single IV injection we found a significantly higher ^198^AuNP fraction in the about 10-fold larger uterus of pregnant rats including the total progeny compared to the rather small uterus of the non-pregnant rats (Figure 2). However, when normalizing the ^198^AuNP content per weight of uterus or blood, the concentrations for a given ^198^AuNP size were similar between pregnant and non-pregnant rats. Yet, there were consistent differences for different ^198^AuNP sizes, Additional file 1: Figure S2; *i.e.*^198^AuNP concentrations in the uterus are mainly determined by the ^198^AuNP concentration in the uterine blood.

**Figure 3:**
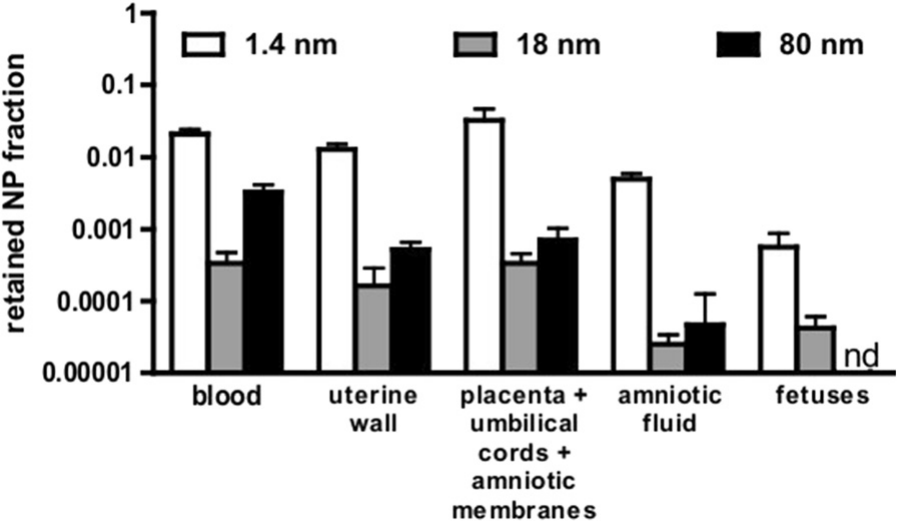
**Twenty-four-hour fractional retention of**^**198**^**AuNP relative to the initially administered dose in blood, uterus and progeny of pregnant rats on day 18 ± 1 of gestation: fractions of**^**198**^**AuNP in blood and the various intra-uterine compartments: total blood, uterine wall, placentas + umbilical cords + amniotic membranes, total amniotic fluid and all fetuses.** (nd = below detection limit of gamma-spectrometer; *i.e.* < 0.3 ng of 80 nm AuNP corresponding to <5 x 10^5^ 80 nm AuNP). Mean values ± SEM are given, n = 4 rats. Results of the statistical ANOVA analysis are given in Table 1.

The translocated ^198^AuNP fractions in the total uterus were 5% of the IV injected 1.4 nm ^198^AuNP and about 0.1% of both 18 nm and 80 nm ^198^AuNP, respectively, Figure 2. This resulted in detectable AuNP-mass-based fetal fractions of 0.0006 and 0.00005 of 1.4 nm and 18 nm ^198^AuNP, respectively, but no 80 nm fraction in fetuses indicating the importance of the AuNP size, Figure 3.

Figure 3 shows retained ^198^AuNP fractions (of the injected ^198^AuNP) in the four compartments of the pregnant uterus ((a) uterine wall, (b) placentas?+?umbilical cords?+?amniotic membranes, (c) total amniotic fluid and (d) all fetuses) 24 h after IV injection of 1.4 nm, 18 nm and 80 nm ^198^AuNP. Results of the statistical analysis are shown in Table 1. Nearly two orders of magnitude higher fractions of 1.4 nm ^198^AuNP were found in the uterine wall or the placentas (compartment b) than for 18 nm or 80 nm ^198^AuNP. In amniotic fluid we found detectable ^198^AuNP fractions of all three sizes. The 1.4 nm ^198^AuNP fraction of 0.005 was 100¿200-fold higher than those detected for the larger ^198^AuNP. There was no significant difference in the amniotic fluid fractions of retained 18 nm and 80 nm ^198^AuNP. Interestingly, 1.4 nm ^198^AuNP concentrations per weight of blood, uterine walls and placenta were significantly (one order of magnitude) higher than those of the larger ^198^AuNP; Additional file 1: Figure S3.

### 8AuNP translocation through the placenta towards the fetus

Figure 3 shows small but detectable amounts of 1.4 nm and 18 nm ^198^AuNP in the fetuses of pregnant rats but none in fetuses treated with 80 nm ^198^AuNP. Only fractions of 0.0006 (30 ng) and 0.00004 (0.1 ng) of 1.4 nm and 18 nm AuNP, respectivel, were detected in the fetuses. Hence, the absence of 80 nm ^198^AuNP demonstrates that even tracer amounts of ^198^AuNP were not translocated by any mechanism consistent with our hypothesis that 80 nm ^198^AuNP are too large to pass transtrophoblastic channels. Our finding that the 80 nm ^198^AuNP could not be observed in the fetus contrasts with those of Yamashita and co-workers [24] who found that 800 ?g i.v. injected 70 nm SiO_2_ NP or 35 nm TiO_2_ NP not only crossed into fetal tissue but also induced significant fetal toxicity. This difference is unlikely due to NP size (70 nm vs. 80 nm) but could be due to either material differences (Au *vs*. SiO_2_ or TiO_2_) or ¿ which we think might be a significant confounder ¿ the non-physiologically high bolus type (800 ?g/mouse IV of SiO_2_ or TiO_2_ NP) delivery which may open normally not functional translocation pathways as it is well known from lung particle overload studies [37]. Regarding the 10-fold difference in weight of rats and mice their NP doses exceeded ours by about a factor of 1000. In contrast, the above mentioned, very recent study by Rattanpinyopituk and coworkers on the translocation of IV injected 20 nm and 50 nm AuNP into the placenta of mice reported only the presence of AuNP in the placenta but not in the fetuses [26]. AuNP sizes are rather similar to our 18 nm and 80 nm AuNP and the administered dose was only 10-fold higher than ours. The absence of AuNP in the fetuses may be related to the lower sensitivity of the chemical Au analysis used (ICP-MS) but may also be affected by possible coagulation of the citrate-stabilized AuNP in saline leading to larger agglomerates which could not cross the placental barrier although immunoreactivity tests suggested that clathrin- and caveolin-mediated endocytosis was upregulated at the maternal¿fetal barrier.

Since we did not find 80 nm ^198^AuNP in fetuses we can conclude: transport of all three AuNP sizes across the placenta by macropinocytosis can be ruled out because all three sizes of ^198^AuNP would likely be taken up in the several-hundred-nm large pinocytotic vesicles independent of the AuNP sizes we used [38]. But we also have to take into account the transport across the placental barrier by receptor-mediated endocytotic processes as the recent study of Rattanapinyopituk and co-workers suggests [26]. Each of our ^198^AuNP will lose the S-TPP coating immediately after IV injection [39] and likely form particle-protein-conjugates with serum proteins shown by results of *in vitro* studies [40]-[42] and as suggested by our preliminary *in vivo* data - see Additional file 1. Formation of particle-protein-conjugates depends on particle size, material and surface properties as well as protein concentrations and their composition in serum [40]. Although the results of the cited reports are based on *in vitro* studies, the formation of a protein corona very likely differs between the three Au NP sizes used as we recently showed by our in vitro protein binding studies using the same AuNP [43]. As a consequence, mechanisms of cell uptake and translocation of the three AuNP across the various hemochorial cell layers can be quite different because receptor mediated endocytosis of particles depends both on particle size and the adsorbed proteins. Indeed, our data are consistent with the notion that 80 nm ^198^AuNP-protein-conjugates are too big for endocytotic transport across the various cell types of the trophoblastic layers into the fetal blood (Figure 1); therefore, no 80 nm AuNP were found in the fetuses. On the other hand we cannot exclude that a fraction of the 18 nm ^198^AuNP found in the fetus was transported *via* endocytosis across the cells of the trophoblastic layers [26], depending on the size and composition of the conjugated protein-layer around the AuNP. For the smallest 1.4 nm ^198^AuNP together with their protein-conjugate endocytotic transport across the trophoblastic layers is most likely, while the passage of the 1.4 nm AuNP through trophoplastic channels is likely to act as an additional and competitive transport across the trophoblastic layers of the placenta.

Because transcellular ^198^AuNP transport from the maternal to the fetal side involves the transport across multiple cell layers of the trophoblast, it likely takes considerably longer compared to a faster passage through a single cell layer and it is also likely to be slower than their passage through transtrophoblastic canaliculi. We base this suggestion on estimates given in the Additional file 1 where we used our earlier *in vivo* translocation data of the same sized ^198^AuNP across the alveolo-capillary-barrier ¿ a membrane consisting of a single cell epithelium, the basal membrane and a single cell endothelium. ¿ Using this comparison, we estimate that the contribution of transcellular 24-h translocation across the trophoblastic layers is a minor fraction of the observed 1.4 nm and 18 nm AuNP in fetuses; see Additional file 1.

### 8AuNP translocation through the amniotic membrane towards the fetus

Production of amniotic fluid in late stage pregnancy is mainly arranged by an inflow of fetal urine and liquid secretion from fetal lungs as well as trans-membranous diffusion across the amniotic membranes, while the main outflow is regulated by fetal swallowing and intra-membranous absorption [34],[44]-[46]. Transdermal uptake by rat fetuses appears to be unlikely in the third trimester of gestation as the skin is already keratinized [31]. The amniotic membranes consist of an epithelial cell layer on a basal membrane and provide a large surface. Hence, ^198^AuNP can reach the amniotic fluids only across these membranes, see Figure 1.

Figure 3 shows the significant, two orders of magnitude higher 1.4 nm ^198^AuNP fraction in the amniotic fluid compared to the amniotic fluid fractions of 18 nm and 80 nm ^198^AuNP. This may be explained by differences in the diffusion of 1.4 nm ^198^AuNP *versus* the larger ^198^AuNP. The 18 nm and 80 nm ^198^AuNP amniotic fractions are not significantly different from each other indicating that their translocation to the amniotic fluid is not due to diffusional transport. Instead, this implies transcellular transport which is believed to be independent of size for 18 nm and 80 nm ^198^AuNP [38]. While the ^198^AuNP concentration per unit mass of blood and amniotic fluid was rather similar for 1.4 nm ^198^AuNP, it was one order of magnitude less for both 18 nm and 80 nm ^198^AuNP in the amniotic fluid than that in blood ¿ see Additional file 1: Figure S3. This difference suggests that diffusion is the predominant transport of the 1.4 nm ^198^AuNP across the amniotic membrane. As stated above, the similarity of 18 nm and 80 nm ^198^AuNP concentrations in the amniotic fluid are consistent with size-independent transcellular transport across the amniotic membrane.

The finding of detectable fractions of all three ^198^AuNP in the amniotic fluid may point to another potential pathway of ^198^AuNP uptake into the fetuses involving swallowing of the amniotic fluid. During normal pregnancy, there is no fecal excretion by the fetus, so the ^198^AuNP are expected to be stored in the fetal gastro-intestinal-tract. Since the fractions of 18 nm and 80 nm ^198^AuNP in the amniotic fluid were similar and fetal swallowing is AuNP size independent one should expect that - if swallowing is the underlying cause for the finding of AuNP in the fetus ¿ the fractions of 18 nm and 80 nm ^198^AuNP in the fetus should be similar and reflecting their concentrations in the amniotic fluid. However, we did not find 80 nm AuNP in the fetuses in contrast to 18 nm AuNP, so we conclude that swallowing of 18 nm or 80 nm ^198^AuNP by the fetuses can be excluded unless the amount being swallowed is below our limit of detection. Consequently, the observed 18 nm ^198^AuNP content in the fetuses results from transfer through the placenta, most likely *via* transtrophoblastic canaliculi although receptor-mediated endocytotic processes described above cannot fully be excluded.

The translocation mechanisms may not necessarily be the same for the 1.4 nm ^198^AuNP found in the fetuses because the amount of 1.4 nm ^198^AuNP in the amniotic fluid is two orders of magnitude higher than those of 18 nm and 80 nm ^198^AuNP; so fetal swallowing may still play a role for these small 1.4 nm AuNP. However, given that the ratio between amounts in the fetal and the placental ^198^AuNP (see Figure 3) are approximately the same for the 18 nm and 1.4 nm ^198^AuNP and given ¿ as explained above - that none of the fetal 18 nm ^198^AuNP is due to swallowing, this implies that only a minimal, if any, amount of the fetal 1.4 nm ^198^AuNP is a result of swallowing.

#### Relevance of the AuNP doses administered to the mother and acuumulated by the fetuses

As mentioned above using the technology of radioactive ^198^Au-labelling of the AuNP it was possible to detect anticipated tracer amounts in the placental and fetal samples of a few ng of ^198^AuNP following IV injection of low mass dose of 5, 3 and 27 ?g/rat of 1.4 nm, 18 nm and 80 nm AuNP, respectively, Table 2. These doses resulted in fetal AuNP mass accumulations of 30 ng and 0.1 ng fractions of 1.4 nm and 18 nm AuNP but none of the 80 nm AuNP. In order to estimate whether the IV administered AuNP doses are relevant to those delivered to the lungs which translocated from the lungs into circulation we compare the data obtained after intratracheal instillation of the same set of AuNP, published previously [43]. There we instilled rather similar AuNP doses intratracheally which are given in Table 3. In addition, the fraction and mass of the translocated AuNP across the air-blood-barrier (ABB) are given. When comparing the AuNP concentrations in blood the doses administered to the pregnant rats they are 25, 1000 and 1500 times higher for 1.4 nm, 18 nm and 80 nm AuNP, respectively, than those of the AuNP which had crossed the ABB. However, also in the previous paper we had aimed to minimize the delivered doses to the lungs of the rats.

**Table 1:** **Statistical ANOVA analysis for Figure**3

**ANOVA analysis**	**AuNP size**	**AuNP size**	**AuNP size**
**Compartm./fluid**	**1.4 vs. 18**	**1.4 vs. 80**	**18 vs. 80**
**Blood**	****	****	ns
**Uterine wall**	****	****	ns
**Placenta + umbilical cords + amniotic membranes**	***	**	ns
**Amniotic fluid**	****	****	ns
**Fetuses**	**	**	ns

**Table 2:** **Parameters and dose metrics of administered**^**198**^**AuNP; additionally**^**198**^**AuNP doses in the fetuses 24 hours after intravenous injection**

AuNPs, core diameter (nm)	1.4	18	80
Hydrodynamic diameter (nm) after neutron irradiation	2.9^#^	21^$^	94^$^
(Ph_2_PC_6_H_4_SO_3_Na); ligand molecules/NP^#^	12	1.5 ¿ 2 x 10^3^^+^	3 - 4 x 10^4+^
Specific ^198^Au radioactivity (GBq/g)	19	31	8.3
Isotope ratio of ^198^Au to stable ^197^Au	4 10^?8^	6 10^?8^	1.2 10^?6^
Ratio of ^198^Au per AuNP	2 10^?6^	1 10^?2^	19
pH Value of suspension	5.6	6.4	5.4
Zeta potential (mV)	?20.0?±?2.4	?22.8?±?3.1	?27.1?±?1.3
Administered mass of AuNP (?g) per rat	5.2?±?0.6	3.2?±?0.9	26.5?±?5.0
Administered number of AuNP per rat	1.9?±?0.2 x 10^14^	5.5?±?1.5 x 10^10^	5.2?±?1.0 x 10^9^
AuNP mass (ng) retained in fetuses	30	0.12	< 0.1*
Number of AuNP retained in fetuses	1.2 x 10^11^	2.4 x 10^6^	< 2 x 10^4^*

**Table 3:** Comparison of intratracheally instilled AuNP dose and the resulting translocated fraction into blood circulation with intravenously injected AuNP doses

AuNP core diameter (nm)	1.4	18	80
IT AuNP dose (?g)	2.6	1.6	17.6
Transloc Fraction	0.08	0.002	0.001
Translocated AuNP mass (?g)	0.208	0.003	0.018
IV AuNP dose pregnant rats (?g)	5.2	3.2	26.5
Dose factor	25	1000	1506

Furthermore, we have performed an inhalation study of freshly generated 20 nm AuNP using the same branch of adult, female rats. This AuNP aerosol was optimized for the highest possible number concentration of about 10^7^ AuNP/cm^3^ being stable for the 5-seconds time between aerosol generation by spark ignition technology and inhalation [49]. This exposure led to an aerosol mass concentration of 1.2 mg/m^3^ due to the high Au density. Using the minute ventilation volume of adult rats of 0.25 L/min [50] and a presumed deposition fraction 0.4 of the inhaled aerosol, then two hours of inhalation are required to deposit about 15 ?g in the rat¿s lungs. This is ten-fold of the intratracheally instilled dose but the AuNP dose which had crossed the ABB would still be two orders of magnitude lower than what had been IV injected to the pregnant rats. For the other two AuNP we don¿t have any aerosol data available but similar relations are expected. However, when screening the current literature on nanomedicinal treatments using AuNP the intravenously injected doses used in experimental animals (mostly mice) are much higher in the range of 1¿10 mg/kg body weight [51]-[55]. In addition, there are a number of preclinical human applications for cancer diagnistics and treatment in which doses of 1¿5 mg/kg BW of superparamagnetic iron oxide NP or have been used. These preclinical studies have been reviewed [56],[57]. Regarding these NP doses in nanomedicinal applications, our AuNP IV doses of 20¿100 ?g/kg BW are very low.

### Extrapolation of AuNP translocation from rat to human placenta

McArdle and coworkers [58] suggest that the transport mechanisms across the placental barrier are similar in species with hemochorial placentas such as rats and humans. Both species have transtrophoblastic channels of about 20¿25 nm diameter [29]-[31] and additional transcellular endocytotic transport mechanisms should be similar. The labyrinth type placenta of rats with a thicker barrier of three trophoblastic layers, *i.e.* more cellular layers than in the human placenta with only one trophoblastic layer, appears to be a conservative model for AuNP transport towards the human fetus. Therefore, we suggest that a similar perhaps even higher AuNP translocation into the human fetus may occur after IV injection.

Indeed, *ex vivo* studies using the human term placenta showed a small but significant translocation of bigger polystyrene particles (from 50 nm up to 240 nm) [22]. Still the highest translocation was found for the smallest NP supporting the importance of NP size but may indicate also some additional processes which may enable NP to cross the human placental barrier in small amounts. Note that the human term placenta after birth is not equivalent to 18-day rat placenta and may already have a compromised barrier function.

Recently, Saunders [35] concluded that currently there is very limited data on the translocation of NP towards the human fetus. Fuchs and co-workers [14] also speculate that endocytotic and transcytotic processes with diffusion, carrier-mediated and vesicular transport are the main mechanisms which transports nutrients like glucose, amino acids, lipids, water, ions, vitamins, minerals and oxygen through the placental barrier. But they also report, that the pathways are poorly or not at all characterized. In addition, the recent study on 20 nm and 50 nm AuNP failed to demonstrate translocation across the placental barrier into the fetuses based on the ICP-MS method used [26].

Similarly, we cannot extrapolate these fractional accumulations in the fetus to other NP contained in consumer products or medication like titania, silica, ceria, silver or carbonaceous NP. Furthermore, any extrapolation to the differential behavior of conventional drugs in pregnant versus non-pregnant rats appears not to be valid since molecules of conventional drugs behave completely different in the organism compared to NP. Yet, the size dependency for translocation may well applicable to other NP materials. So, while these results suggest that the fetus is well protected against larger NP the unborne may well be exposed to very small NP during the mother¿s pregnancy through medical treatment or via food consumption.

## Conclusions

In conclusion, our study design and results allowed to differentiate between two potential ^198^AuNP pathways from maternal blood to the fetus in a pregnant rat model at gestation day 18 ¿ (1) *via* placenta by transtrophoblastic channels competing with transcellular endocytotic passage across multiple cell layers and (2) *via* transport across the amniotic membrane ¿ both pathways are ^198^AuNP size dependent:

 The absence of 80 nm ^198^AuNP in the fetuses is consistent with our hypothesis that these AuNP are too large to pass the ~20-25 nm sized transtrophoblastic channels.

 We infer from our results that both 1.4 nm and 18 nm ^198^AuNP are transported through the placenta ¿ *via* transtrophoblastic channels and/or transcellular receptor-mediated endocytotic mechanisms. (Note that this study did not allow a clear distinction between these two transport pathways)

 All three ^198^AuNP sizes cross the amniotic membrane: the 1.4 nm ^198^AuNP by diffusion and/or transcellular transport but the 18 nm and 80 nm ^198^AuNP mainly by the latter transport and are detectable in the amniotic fluid but they are not incorporated into the fetuses within 24 hours.

Therefore, our overall conclusion is that translocation through transtrophoblastic channels is the dominating pathway for ^198^AuNP smaller than the channel diameter of about 20¿25 nm. Furthermore, we suggest that these results can be extrapolated to humans because of the similarity between human and rat late-term placenta.

## Methods

Sulfonated triphenylphosphine (S-TPP) coated AuNP of 1.4 nm, 18 nm and 80 nm core diameter were synthesized following known procedures [59],[60]. While 1.4 nm AuNP were ideally monodisperse, the standard deviation of both 18 nm and 80 nm AuNP was about 10% in the distilled water suspension, see Table 2. All AuNP were radio-labeled with ^198^Au by neutron activation at a neutron flux of 10^14^ cm^?2^ sec^?1^ in the research reactor of Helmholtz Center Berlin, Germany (^198^Au half-life 2.69 d; 411 keV gamma emission used for gammaspectroscopic analysis). Gold amounts and irradiation times were adjusted to provide sufficient ^198^Au radioactivity for the subsequent *in vivo* studies. Specific ^198^Au radioactivity and the isotope ratio of ^198^Au to stable ^197^Au are given in Table 2. Note that this ratio is very low such that statistically only one ^198^Au isotope can be found in a 1.4 nm and 18 nm AuNP and most of the 1.4 nm AuNP do not contain any ^198^Au atom at all while there are fewer 18 nm AuNP containing no ^198^Au isotope; but in the 80 nm AuNP an average number of 20 ^198^Au atoms are contained in the AuNP matrix, Table 2.

After neutron irradiation immediately prior to rat application the 1.4 nm ^198^AuNP solution was filtered through a 10 cm column of Celite to remove agglomerates; losses determined by ^198^Au radioactivity accounted for about 10% [10]. The 18 nm and 80 nm ^198^AuNP suspensions were visually controlled for precipitates and their correct pink translucent color of the colloidal suspension immediately prior to the application in rats; no change in color or precipitation and no changes were found compared to the suspension prior to irradiation. In case of 18 nm and 80 nm ^198^AuNP their UV absorption peak at 523 nm was unchanged prior to and three weeks after irradiation (data not shown). The hydrodynamic diameters (HD) of the 18 nm and 80 nm AuNP were measured in duplicate by photon correlation spectroscopy (PCS; Malvern HPPS5001, Herrenberg, Germany). The HD were slightly increased to 21 nm and 85 nm (polydispersity index 0.18) according to the S-TPP coating ¿ see Table 2 - and a very small fraction of agglomerates (when AuNP volume and not the intensity was plotted the fraction of agglomerates disappeared; data not shown). Zeta potential of the radiolabeled ^198^AuNP was measured in a distilled water suspention as used for rat application; 15 cycles, 10 runs, for each sample in triplicate (ZetaPals, Brookhaven Instruments). For other AuNP parameters see Table 2. The 1.4 nm AuNP solution and the 18 nm AuNP suspensions remained stable during at least two weeks without any detectable precipitation or change of color. Due to gravitational sedimentation 80 nm AuNP settled during two weeks but could be re-dispersed by vortexing into the same pink suspension as before.

### Animals

Twenty-four healthy, adult female Wistar-Kyoto rats (WKY/Kyo@Rj rats, Janvier, Le Genest Saint Isle, France), 3¿4 months of age and about 250 g body weight (BW) prior to pregnancy,) were used in these studies; twelve of which were pregnant and were enclosed into the experimental protocol on day 18?±?1 of gestation (3^rd^ trimester); All rats were housed in pairs in humidity- and temperature-controlled ventilated cages on a 12 h day/night cycle prior to the experiments. A rodent diet and water were provided *ad libitum*. Groups of four pregnant rats or four non-pregnant controls were randomly assigned to the IV administration of the three different-sized ^198^AuNP. The *in-vivo* biodistribution studies were conducted under German federal guidelines for the use and care of laboratory animals and were approved by the Regierung von Oberbayern (Government of District of Upper Bavaria, Approval No. 211-2531-94/04) and by the Institutional Animal Care and Use Committee of the Helmholtz Zentrum München - German Research Center for Environmental Health.

### 8AuNP administration and analysis of 198AuNP biodistribution

Colloidal suspensions of 1.4, 18 or 80 nm ^198^AuNP were slowly injected into the tail vein of pregnant rats or non-pregnant controls [4],[10]. For 1.4 nm and 18 nm ^198^AuNP doses of about 5 ?g per rat were chosen and 25 ?g per rat of the 80 nm ^198^AuNP, respectively. Rats were anesthetized by inhalation of 3-5% isoflurane until muscular tonus relaxed. A suspension volume of 130 ?L containing ^198^AuNP was placed at the lower end of a 1-mL-insulin-syringe without any air at the tip of the syringe. A flexible intravenous catheter (diameter 24G) was placed into the tail vein. Initially, 100 ?L phosphate buffered saline (PBS) was injected testing for controlling adequate positioning of the catheter in the tail vein before the syringe with the ^198^AuNP suspension was connected and the suspension was slowly injected during about 30 seconds. The dead space in the syringe and connector had been determined to be 80 ?L such that a dose of 50 ?L ^198^AuNP suspension was injected into the tail vein. In Table 2 the ^198^AuNP doses in terms of gold mass, surface and numbers are given for all three sizes.

Twenty-four hours after ^198^AuNP administration the rats were killed by exsanguinations cannulating the abdominal aorta and aspirating blood with a syringe under deep anesthesia by continuous isoflurane inhalation (3-5%) until death. About 70% of the blood volume was sampled *via* the abdominal aorta as estimated from the blood volume and BW. As described earlier [61],[62] all organs including the uterus with all amnions and fetuses and tissues of interest, the entire remaining carcass and the total excreta during 24 hours were weighed in wet state and stored for radio-analysis. While non-pregnant controls were housed singly in metabolic cages and urinary and fecal excreta were collected separately, the pregnant rats were kept in normal cages to avoid any stress and fecal droppings were manually separated from the bedding with the urine for separate radio-analysis. To avoid any cross contamination, no organs were cut open and all body fluids were sampled immediately *via* cannulation of vessels or excretory ducts before cutting. Hence, the following samples were radio-analyzed:

 Uterus: (a) for non-pregnant rats the uterus was one sample; (b) for pregnant rats there were four compartments: (i) the uterine wall, (ii) the placentas together with all umbilical cords and amniotic sacs, (iii) the total of all amniotic fluids which were collected by cannulating each amnion and (iv) the total of all fetuses; these four compartments of each rat were radio-analyzed.

 Other organs: lungs, liver, spleen, kidneys, brain, heart, total exsanguinated blood, gastro-intestinal tract (GIT) including: esophagus, stomach, small and large intestine;

 Tissues: total skin, sample of muscle, sample of bone: femur; the injection site of the tail was separated;

 Remainder: total remaining carcass beyond the listed organs and tissues;

 Excretion: total urine and feces, collected separately.

Without any additional preparatory step all samples were radio-analyzed for ^198^Au content.

### A complete balance of 198Au radioactivity

A complete balance of ^198^Au radioactivity retained in the body and cleared by excretion out of the body was quantified by gamma-spectroscopy in either a 10-mL-well-type NaI(Tl) scintillation detector for small samples (<3 g) or a 1-L-well-type NaI(Tl) scintillation detector for large samples like the remaining carcass [61],[63] thoroughly lead-shielded for reduction of background radiation. From measured count rates, corrected for background and radioactive decay and calibrated with a well-defined ^198^Au source, amounts of radioactivity at reference date were calculated. Samples yielding net counts (*i.e.* background-corrected counts) in the photo-peak region-of-interest of the ^198^Au gamma spectrum were defined to be below the detection limit when they were less than three standard deviations of the background counts of this region-of-interest. Therefore, calculated amounts of radioactivity are directly proportional to the mass of ^198^AuNP. The sum of all ^198^Au amounts of radioactivity was compared to the administered dose as determined by radio analysis of an aliquot of the administered ^198^AuNP solution. Hence, total radioactivity equals the administered radioactivity per rat to which ^198^Au radioactivity of each sample was normalized as a fraction.

### Statistical analysis

For statistical data analysis Graph pad prism 4.0 was used. All calculated significances are based on a one-way analysis of variance (ANOVA) followed either by a post hoc Tukey test or post hoc Sidak test as indicated in the Figure legends. In case of an individual two-group comparison, the unpaired t test was used.

## Competing interests

All authors declare no competing financial interests.

## Authors¿ contributions

Study design: MSB, WGK, GO. Study performance and AuNP preparation: MSB, WGK, GS, JL, AW, MS. Data evaluation and analysis including statistics: MSB, WGK, AW, MS, FT, SH. Manuscript editing: MSB, WGK, GO, MS, FT, JL, GS. All authors read and approved the final manuscript.

## Authors¿ information

MSB: ^5^Current address: Bavarian Health and Food Safety Authority, 85764 Oberschleissheim, Germany.

SH: ^6^Current address: Walter Brendel Centre of Experimental Medicine, Ludwig-Maximilians-Universität München, Munich, Germany.

FT: ^7^ Current address: FOCAS institute, Dublin Institute of Technology, Dublin, Irland.

## Additional file

## Supplementary Material

Additional file 1: Characterization of the physico-chemical parameters. Click here for file
