# Supplementary material for: Size dependent translocation and fetal accumulation of gold nanoparticles from maternal blood in the rat
Source: Part Fibre Toxicol. 2014 Sep 10;11:33. doi: 10.1186/s12989-014-0033-9 (PMC4445676; doi:10.1186/s12989-014-0033-9)
Supplement: Additional file 1: — Characterization of the physico-chemical parameters. [file s12989-014-0033-9-S1.pdf]

Supplementary information

Size dependent translocation and fetal accumulation of gold nanoparticles from maternal blood in the rat

Manuela Semmler-Behnke[5,1], Jens Lipka[1], Alexander Wenk[1], Stephanie Hirn[6,1], Martin Schäffler[1], Furong Tian[7,1], Günter Schmid[3], Günter Oberdörster[2] and Wolfgang G Kreyling[1,4]

Characterization of the physico-chemical parameters

The sulfonated triphenylphosphine (S-TPP) surface functionality of the ligand protected AuNP are characterized by the high mobility of the S-TPP molecules on the AuNP surface. As it is known, they can easily be substituted by stronger ligands, following the rules of complex chemistry [1]. In a biological system, numerous stronger ligands such as proteins, sulfur containing molecules *etc*. are available and will substitute the original phosphines partially or quantitatively. Hence, the administration of initially S-TPP-coated AuNP provides binding dynamics of serum proteins and biomolecules in blood to the naked surface of the injected AuNP after the phosphine ligand has been replaced. Note that the free S-TPP did not cause any detectable toxic response in *in vitro* tests [2]. Our latest *in vivo* AuNP inhalation studies support the notion of the rapid replacement of the S-TPP ligand coating in the lungs: When comparing the 24 hours biodistribution after intratracheal instillation of 18 nm AuNP with that of freshly generated, pristine 20 nm AuNP inhaled as an aerosol, the biodistribution in all organs, blood, remaining carcass and in excretion was remarkably similar suggesting rapid removal of the S-TPP coating and similar patterns of lung protein binding after both applications.

In Figure S1a+b the hydrodynamic size distributions (volume, dynamic light scattering measurements by HPSS, Malvern, Herrenberg, Germany) are shown for original and neutron-irradiated 18 nm and 80 nm AuNP coated with S-TPP. The hydrodynamic size distribution measurement of 1.4 nm AuNP were not possible using dynamic light scattering.

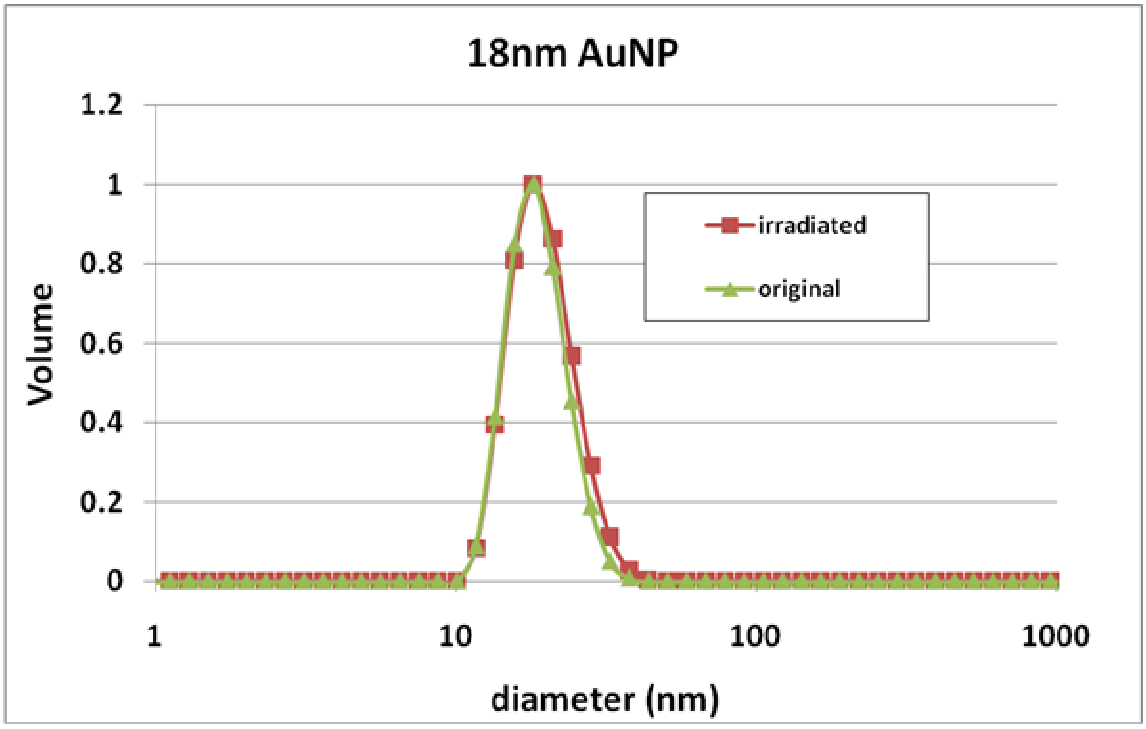

Figure S1a: Hydrodynamic size distribution (volume) of original and neutron-irradiated 18 nm AuNP coated with sulfonated triphenylphosphine; Z_{avg} is 21 nm and 22 nm, respectively; PdI is 0.07 and 0.14, respectively.

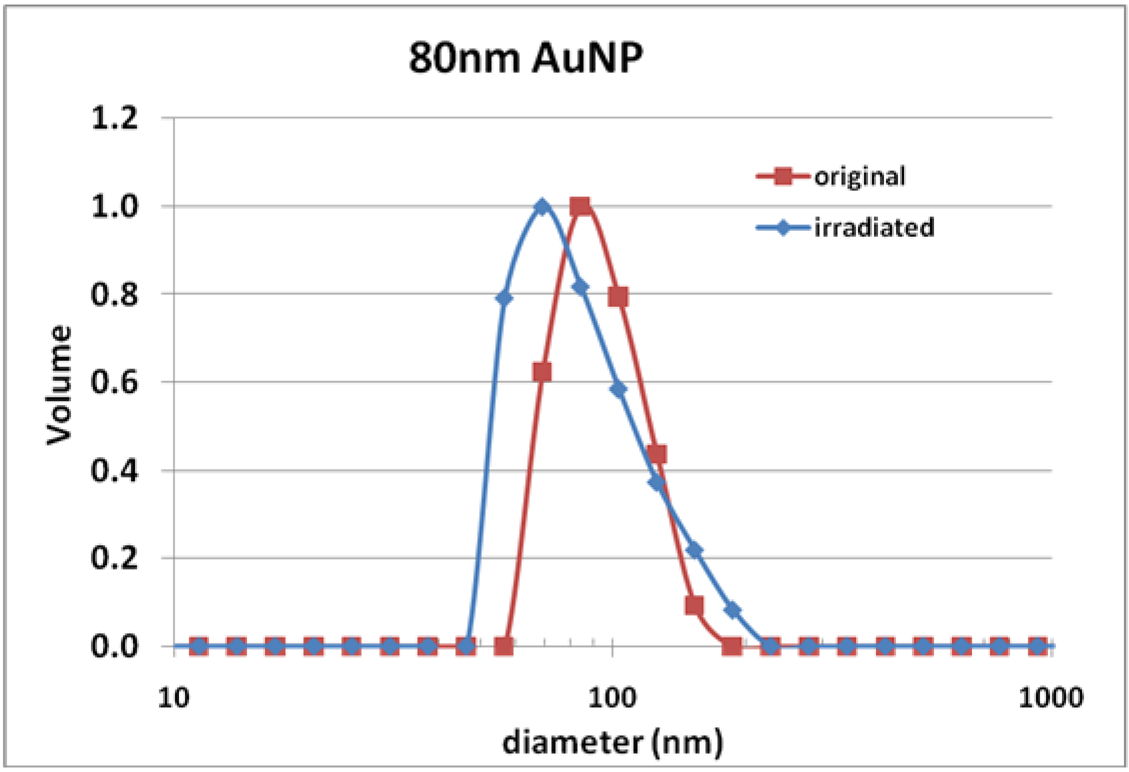

Figure S1b: Hydrodynamic size distribution (volume) of original and neutron-irradiated 80 nm AuNP coated with sulfonated triphenylphosphine; Z_{avg} is 89 nm and 94 nm, respectively; PdI is 0.15 and 0.12, respectively.

AuNP concentrations per mass of organs and tissues

In Figure S2 the retained ^{198}AuNP fractions of the uterus and blood of pregnant rats *versus* non-pregnant controls are given as concentrations (fractions per weight of uterus or blood) for all three ^{198}AuNP sizes. Au-mass concentrations (in µg) are obtained when multiplying these fractions with the IV administered AuNP masses given in Table 2. Concentrations of 1.4 nm ^{198}AuNP are about one order of magnitude higher than those of 18 and 80 nm ^{198}AuNP. Note the similarity of concentrations in the uterus of both pregnant rats and non-pregnant controls for each given ^{198}AuNP size; the same is observed for ^{198}AuNP concentrations in blood indicating that the ^{198}AuNP concentrations in the uterus of both pregnant rats and non-pregnant controls are mainly determined by the ^{198}AuNP concentration of the remaining blood volume of the uterus.

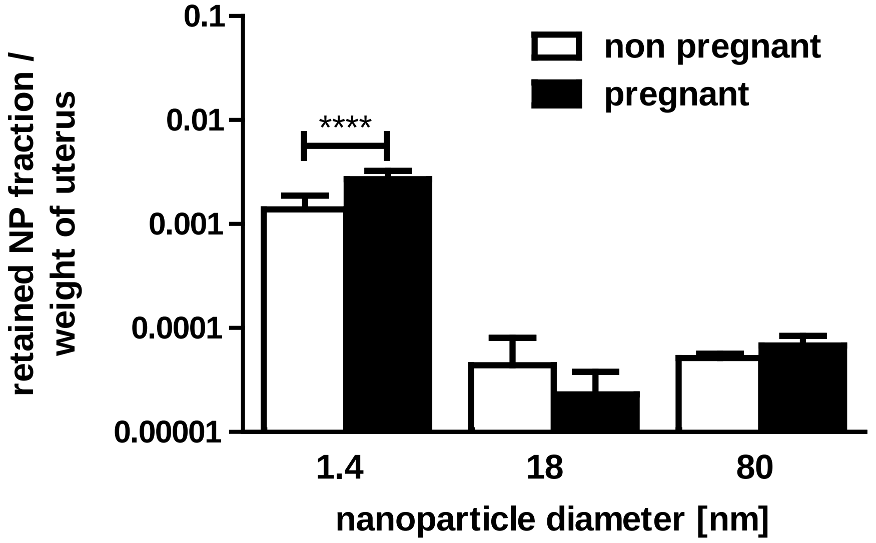

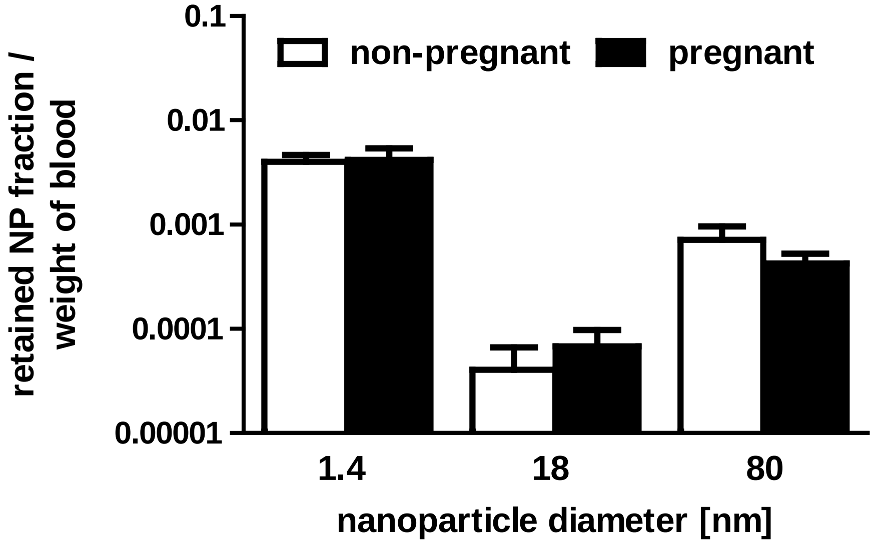

Figure S2. Fractional concentrations of ^{198}AuNP (relative to the initially administered dose) in uterus (upper panel) and blood (lower panel) of pregnant *versus* non-pregnant rats: Retained ^{198}AuNP fractions per weight of uterus or blood of both pregnant rats and non-pregnant controls. For each ^{198}AuNP size the concentrations are rather similar between pregnant rats and non-pregnant controls. (n=4). Au-mass concentrations (in µg) are obtained when multiplying these fractions with the IV administered AuNP masses given in Table 1. (n=4; **** p < =0.0001). Statistical analysis of pregnant vs. non-pregnant rats by one-way analysis of variance (ANOVA) followed by post hoc Sidak's multiple comparisons test.

In Figure S3 the retained ^{198}AuNP fractions in blood and the various compartments of the uterus of pregnant rats are given as ^{198}AuNP concentrations (fractions per organ weight). Note the similarity of 1.4 nm ^{198}AuNP concentrations in blood, uterine wall and placenta; the same holds for 18 nm ^{198}AuNP. This suggests predominant AuNP retention in blood of the uterine walls and the placenta. The concentration of 80 nm ^{198}AuNP in blood is about a factor of 5 higher than in the uterine wall and in the placenta compartment. Furthermore, while the ^{198}AuNP concentration per weight of blood and amniotic fluid was rather similar for 1.4 nm ^{198}AuNP, it was one order of magnitude less in the amniotic fluid for both 18 nm and 80 nm ^{198}AuNP than in blood. This difference suggests diffusion is the predominant transport mechanism of the 1.4 nm ^{198}AuNP across the amniotic membrane; and likewise the concentration gradient of 18 nm and 80 nm ^{198}AuNP in the amniotic fluid may indicate transcellular transport across the amniotic membrane.

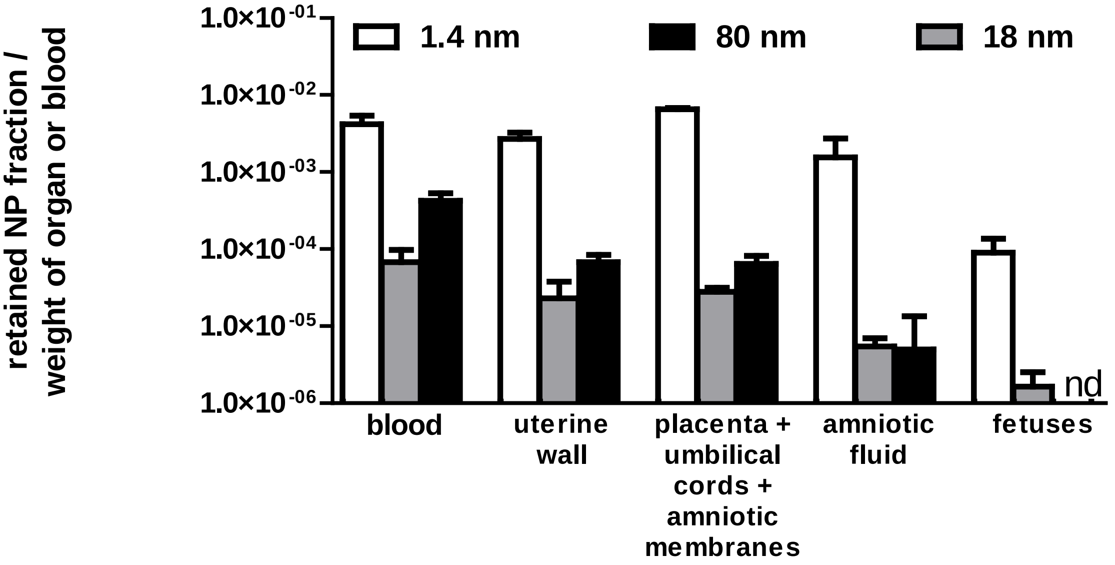

ANOVA analysis	AuNP size	AuNP size	AuNP size
Compartm. / fluid	1.4 vs. 18	1.4 vs. 80	18 vs. 80
blood	****	****	ns
uterine wall	****	****	ns
placenta + umbilical cords + amniotic membranes	****	****	ns
amniotic fluid	*	*	ns
fetuses	**	**	ns

Figure S3. Retained NP fractions per weight of uterus compartment or blood in pregnant rats. ^{198}Au radioactivity- and, hence, Au-mass-based fractions are normalized to the initially administered dose. Compartments of the uterus are: (a) uterine walls, (b) placenta compartment consisting of placentas, umbilical cords and amniotic sacs, (c) total amniotic fluids, (d) all fetuses. (n=4; nd = below detection limit; * $p < 0.05$; ** $p < 0.01$; *** $p < 0.001$; **** p < =0.0001). Au-mass concentrations (in µg) are obtained when multiplying these fractions with the IV administered AuNP masses given in Table 1. Results of statistical analysis

by one-way analysis of variance (ANOVA) followed by post hoc Tukey's multiple comparisons test are given in the table of this legend.

AuNP elimination from blood

Regarding the measurements of AUC after an IV bolus injection of AuNP we have analyzed the biodistribution at two time points (1h, 24h) after IV injection which is published in a previous paper [3]. We now are comparing the total AuNP contents in blood at two time points (1h, 24h) of non-pregnant rats with the data of this MS.

Table S1: Estimated AuNP content (%) in total blood 1 h and 24 h after IV injection

AuNP core diameter (nm)	1,4	18	80
	mean ± std	mean ± std	mean ± std
1h IV injection	53.95 ± 7.01	0.14 ± 0.03	0.74 ± 0.27
24h IV injection	6.76 ± 0.61	0.11 ± 0.05	0.30 ± 0.12

Table S1 from the previous paper [3] shows that about half of the 1.4 nm AuNP were still circulating in blood one hour after IV injection. This high level decreased by a factor of 10 within the next 23 h such that still 7 % were circulating. About 99% of the 18 nm AuNP have been eliminated from blood within 1 h and this level was found also after 24 h. Surprisingly, a five-fold higher concentration of the 80 nm AuNP were left in blood after 1 h which declined to about half during 24 h after injection. Since the AuNP concentrations in blood were reproduced rather well by non-pregnant rats of the current manuscript (Figure 3) and, in addition, there is very little difference between pregnant and non-pregnant rats as shown in Figures 3 and S2, the assumption is plausible that similar data after 1 h as those in Table 3 are to be expected in pregnant rats 1 h after injection. So the elimination kinetics from blood to the organs and tissues within 24 h after injection is similar between pregnant and non-pregnant rats for the same sized AuNP but differs largely between the three different AuNP sizes.

Hepato-biliary clearance (HBC) of AuNP

In both IV injection studies, [3] and the current, fecal excretion results predominantly from hepato-biliary AuNP clearance. The kinetics data of hepato-biliary clearance (HBC, i.e. AuNP in GIT + feces) after 1 h and 24 h of the previous paper [3] are shown in the Table S2 below:

Table S2: Hepato-biliary clearance (%) expressed as the sum of GIT content and cumulative fecal excretion 1 h and 24 h after IV injection as previously described [3]

AuNP core diameter (nm)	1,4	18	80
	mean $\pm$ std	mean $\pm$ std	mean $\pm$ std
1h IV injection	2.85 $\pm$ 0.07	0.58 $\pm$ 0.25	0.11 $\pm$ 0.03
24h IV injection	4.60 $\pm$ 0.75	0.51 $\pm$ 0.21	0.33 $\pm$ 0.09

A dominant part of the HBC is already occurring during the first hour after IV injection. Whether this rapid kinetics also corresponds in pregnant rats, remains to be an open question.

The fecal excretion of all three AuNP is dominant compared to urinary excretion but differs significantly not only between the three AuNP sizes but also between pregnant and non-pregnant rats.

In Figure S4 the total fractional excretion of ^{198}AuNP within 24 h after administration is shown in pregnant rats (18±1 d of gestation) and non pregnant controls for three ^{198}AuNP sizes. Excretion includes the ^{198}AuNP contents of the gastro-intestinal-tract (GIT) and feces as well as urine. The GIT and fecal excretion represent hepato-biliary clearance of ^{198}AuNP [3]. While urinary excretion of 1.4 nm ^{198}AuNP is measurable and slightly lower (0.03) in pregnant rats compared to non-pregnant controls (0.06) , urinary excretions of the larger ^{198}AuNP are negligible. Hence, for 18 nm and 80 nm ^{198}AuNP, excretion is dominated by hepato-biliary clearance of ^{198}AuNP. For 1.4 nm ^{198}AuNP HBC is significantly higher than that of 18 nm ^{198}AuNP and HBC is even signifcantly lower for the 80 nm ^{198}AuNP. Hence, HBC differs significantly not only between the three AuNP sizes but also between pregnant and non-pregnant rats. .

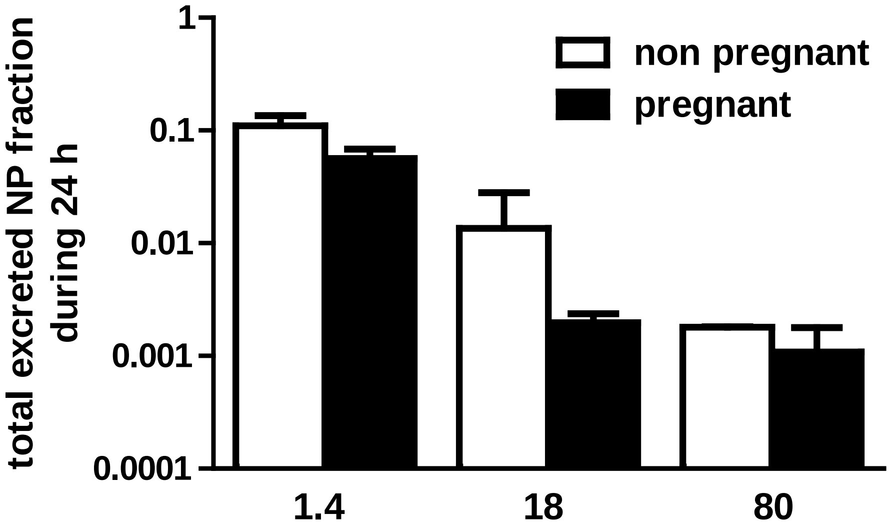

ANOVA analysis	Preg.	Non-preg.	Pregn. vs. non-preg.
**AuNP size**			
**1.4**			***
**18**			ns
**80**			ns
**1.4 vs. 18**	**	****	
**1.4 vs. 80**	**	****	
**18 vs. 80**	ns	ns	

Figure S4: Total fractional excretion of ^{198}AuNP within 24 h after administration in pregnant rats (18±1 d of gestation) and non-pregnant controls for three AuNP sizes. Fractional excretion is normalized to the initially administered dose. Horizontal bars indicate significant changes of corresponding bars below. (n=4; * $p < 0.05$; ** $p < 0.01$; *** $p < 0.001$; **** p < =0.0001). Results of statistical analysis by one-way analysis of variance (ANOVA) followed by post hoc Sidak's multiple comparisons test are given in the table of this legend.

The rapidly decreasing HBC with increasing AuNP size had already been shown in our previous paper [3] using an even larger set of these AuNP. As noted there, NP in the liver,– not immediately trapped by Kupffer cells – are translocated through the fenestrated vascular endothelium into the Dissé spaces to be taken up by hepatocytes and processed into biliary canaliculi [4]. From there, they are drained via the biliary duct to the beginning of the small intestine where they become included in the fecal excretion. It remains to be determined in the future which of the mechanisms involved cause the strong AuNP size dependency observed.

During late-stage pregnancy numerous physiological processes are changed compared to the non-pregnant rat, like a 10-fold increase in the volume of the uterus, significant changes in the blood flow and nutritional contents in the blood for fetal supplement, functional alterations including the role of altered hormone levels, etc. These changes potentially affected the different HBC patterns observed

between pregnant and non-pregnant rats. The reduced HBC occurs simultaneously with a higher AuNP retention in liver during pregnancy (Figure 2); yet, the underlying mechanisms, particularly, in pregnancy are not yet understood and require further investigations. Since very little is known about HBC of different NP and their physico-chemical properties and holds also for nanostructured drugs, no rational comparisons or extrapolations can be drawn currently. Our data provide a first attempt to shed light on the physiological function of HBC including the differences between pregnancy and non-pregnancy.

Estimate of translocated AuNP rate across the placental barrier by transcytosis

Rather than estimating AuNP translocation rates through an *in vitro* monolayered, epithelial cell culture [5] or similar, we base this estimate on our previous *in vivo* translocation data across the alveolar-capillary-barrier of the alveolar region of rat lungs of the same Wistar-Kyoto strain for which we have determined data obtained for the same 1.4 nm and 18 nm ^{198}AuNP that were used in the present paper [6]. Since in the present paper no 80 nm ^{198}AuNP were found in the fetuses, transcytosis – if it did occur – must have been below the detection limit. The lung alveolar-capillary-barrier consists of a single layer of epithelial cells, a basal membrane and the single layer of endothelial cells and does not have canaliculi [7]. The placenta also consists of a maternal epithelial layer, a basal membrane and the fetal endothelial layer but the epithelium consists of multiple layers of syncytiotrophoblastic cells through which transcytosis would have to occur to reach the fetal blood circulation. Furthermore, in both the placental and the alveolar-capillary-barrier tight junctions between epithelial cells are reported [7]. Therefore, we suggest that the alveolar-capillary-barrier may be viewed as a model barrier for AuNP translocation kinetics across barriers. Because the single cell layers in the lungs are much thinner than in the placenta the translocation rate through the alveolar-capillary-barrier may be considered an overestimate of AuNP transcytosis across the placenta. For the rat lungs we determined 24-hours translocation rates towards blood circulation of 0.084 and 0.0020 d^{-1} of the intratracheally instilled 1.4 and 18 nm ^{198}AuNP, respectively [6].

A rough estimate of 1.4 nm and 18 nm ^{198}AuNP transfer rates from the placenta to the fetus can be obtained from the ratios of 0.017, and 0.24 d^{-1} between ^{198}AuNP fractions in fetuses and placenta of Figure, respectively. The translocation rate (0.0020 d^{-1}) of the 18 nm ^{198}AuNP across the lung barrier is much lower than the placental rate (0.24 d^{-1}) indicating not an over-estimate, but the opposite. This implies that the placental transcellular pathway is negligible and suggests a dominant transport through another route such as the transtrophoblastic canaliculi. For the 1.4 nm ^{198}AuNP the transfer rate across the lung barrier is a factor of 5 higher than for the placenta supporting the notion that

according to the lower transfer rate, a small fraction of the 1.4 nm ^{198}AuNP may have crossed the placental barrier by transcytotic processes. However, additional differences in transfer mechanisms of the 1.4 nm ^{198}AuNP between alveolar and placental barriers need to be considered: while fast diffusion across the thin alveolar-capillary-barrier may occur, an equally fast translocation through the transtrophoblastic channels of the thicker trophoblast may result in the above mentioned five-times smaller placental transfer rate. In fact, fast diffusion and cellular uptake in the alveolar epithelium has indeed been reported by Geiser and co-workers [8].

References

1. Pan Y, Leifert A, Ruau D, Neuss S, Bornemann J, Schmid G, Brandau W, Simon U, Jahnen-Dechent W: **Gold Nanoparticles of Diameter 1.4 nm Trigger Necrosis by Oxidative Stress and Mitochondrial Damage.** *Small* 2009, **5:**2067-2076.
2. Tsoli M, Kuhn H, Brandau W, Esche H, Schmid G: **Cellular uptake and toxicity of Au55 clusters.** *Small* 2005, **1:**841-844.
3. Hirn S, Semmler-Behnke M, Schleh C, Wenk A, Lipka J, Schaffler M, Takenaka S, Moller W, Schmid G, Simon U, Kreyling WG: **Particle size-dependent and surface charge-dependent biodistribution of gold nanoparticles after intravenous administration.** *Eur J Pharm Biopharm* 2010.
4. Johnston HJ, Hutchison G, Christensen FM, Peters S, Hankin S, Stone V: **A review of the in vivo and in vitro toxicity of silver and gold particulates: particle attributes and biological mechanisms responsible for the observed toxicity.** *Critical Reviews in Toxicology* 2010, **40:**328-346.
5. Yacobi NR, Demaio L, Xie J, Hamm-Alvarez SF, Borok Z, Kim KJ, Crandall ED: **Polystyrene nanoparticle trafficking across alveolar epithelium.** *Nanomedicine* 2008, **4:**139-145.
6. Semmler-Behnke M, Kreyling WG, Lipka J, Fertsch S, Wenk A, Takenaka S, Schmid G, Brandau W: **Biodistribution of 1.4- and 18-nm gold particles in rats.** *Small* 2008, **4:**2108-2111.
7. Stone KC, Mercer RR, Gehr P, Stockstill B, Crapo JD: **Allometric relationships of cell numbers and size in the mammalian lung.** *American Journal of Respiratory Cell and Molecular Biology* 1992, **6:**235-243.
8. Geiser M, Rothen-Rutishauser B, Kapp N, Schurch S, Kreyling W, Schulz H, Semmler M, Im Hof V, Heyder J, Gehr P: **Ultrafine particles cross cellular membranes by nonphagocytic mechanisms in lungs and in cultured cells.** *Environmental Health Perspectives* 2005, **113:**1555-1560.
